# Identifying Adolescent Mental Health and Behavioral Risk Profiles: The Role of Childhood Adversities, Family Environment, and Peer Support

**DOI:** 10.1002/brb3.71531

**Published:** 2026-06-08

**Authors:** Firoj Al‐Mamun, Moneerah Mohammad ALmerab, Mohammed A. Mamun

**Affiliations:** ^1^ CHINTA Research Bangladesh, Savar Dhaka Bangladesh; ^2^ Department of Public Health University of South Asia Dhaka Bangladesh; ^3^ Department of Psychology College of Education and Human Development Princess Nourah Bint Abdulrahman University Riyadh Saudi Arabia

**Keywords:** adolescents, Bangladesh, childhood adversity, latent class analysis, mental health, peer support

## Abstract

**Background:**

Adolescents in low‐ and middle‐income countries (LMICs) experience a disproportionately high burden of emotional and behavioral problems (EBPs), shaped by early adversities, limited psychosocial support, and structural disadvantages. In Bangladesh, mental health concerns among adolescents remain under‐recognized, with limited data on how mental health symptoms cluster and which subgroups are at elevated risk. Identifying distinct risk profiles is essential for informing targeted interventions.

**Methods:**

Using data from a cross‐sectional school‐based survey (*N* = 1040), we applied latent class analysis (LCA) to identify mental health risk profiles based on emotional symptoms, conduct problems, insomnia, suicidal behavior, truancy, physical fights, bullying, and peer/social isolation. Multinomial logistic regression models were used to examine associations between latent class membership and adverse childhood experiences (ACEs), family/peer relational factors, and sociodemographic characteristics.

**Results:**

Three distinct mental health and behavioral risk classes were identified: low risk (36.3%), moderate risk (36.1%), and high risk (27.6%). The high‐risk class showed elevated internalizing symptoms, externalizing behaviors, and psychosocial adversities, including conduct problems, loneliness, truancy, bullying, and physical fighting. In the fully adjusted model, sexual abuse (aOR = 2.64) and witnessing violence aganist the mother (aOR = 3.56) were the strongest predictors of high‐risk membership. Lack of peer support was significantly associated with both moderate‐risk (aOR = 2.13) and high‐risk (aOR = 2.25) membership, whereas poor parental understanding increased the odds of moderate‐risk classification (aOR = 1.70). In the interaction model, females exposed to household mental illness had disproportionately higher odds of belonging to the high‐risk class.

**Conclusion:**

Adolescent mental health in Bangladesh is shaped by a complex interplay of trauma, family functioning, and gender. Trauma‐informed, gender‐sensitive interventions are urgently needed to address these disparities.

## Graphical abstract

Latent class analysis identified three distinct mental health and behavioral risk profiles among Bangladeshi adolescents. Childhood adversities, poor parental understanding, and lack of peer support increased vulnerability to high‐risk profiles, whereas females exposed to household mental illness showed disproportionately elevated risk, highlighting the need for trauma‐informed and gender‐sensitive interventions.

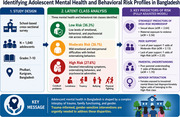

## 1 Introduction

Adolescence is a critical developmental period marked by rapid biological, psychological, and social transitions that significantly shape mental health trajectories across the life course (Patton et al. 2016). Globally, mental health disorders are among the leading causes of disease burden in adolescents, accounting for 13% of the total disease burden (Kieling et al. 2024). These challenges are particularly acute in low‐ and middle‐income countries (LMICs), where adolescents are disproportionately exposed to structural inequalities, environmental adversities, and limited access to mental health care (Hughes et al. 2017; Rathod et al. 2017).

In Bangladesh, adolescent mental health has emerged as an urgent yet under‐recognized public health concern. A growing body of evidence indicates that a substantial proportion of adolescents experience emotional distress, behavioral disorders, and suicidal ideation (Al‐Mamun, Islam, et al. 2024; Al‐Mamun, Al Habib, Islam, et al. 2025). Despite these alarming trends, mental health remains inadequately prioritized in national health policies, and culturally appropriate screening tools or intervention programs are scarce. Many adolescents in Bangladesh are raised in socioeconomically disadvantaged households and are frequently exposed to adverse childhood experiences (ACEs), including physical and emotional abuse, household violence, and parental mental illness—each of which has been strongly associated with poor psychological outcomes (Felitti et al. 1998; Hughes et al. 2017). In addition to ACEs, the quality of family and peer relationships plays a crucial role in shaping adolescent mental health. Limited family support, peer rejection, and a lack of adult guidance in home and school settings have all been shown to elevate psychological risks, especially among females (Butler et al. 2022). Conversely, the presence of supportive relationships across multiple domains serves as a protective factor, promoting emotional resilience and psychological well‐being (Butler et al. 2022; Demetriou 2025).

Furthermore, sociodemographic and psychosocial factors interact with these experiences to influence mental health outcomes. Gender differences are well‐documented: Females are more prone to internalizing symptoms such as emotional distress, whereas males are more likely to exhibit externalizing behaviors, including aggression and peer‐related difficulties (Al‐Mamun, Islam, et al. 2024). Contextual variables such as parental education, household income, and urban–rural residence intersect with school climate, peer interactions, and family functioning to create complex patterns of risk and resilience (Al‐Mamun, Islam, et al. 2024; Sheikh et al. 2016). These intersecting dimensions highlight the need for integrative research approaches that can account for the multifactorial nature of adolescent mental health.

Traditional approaches to assessing adolescent mental health have largely relied on variable‐centered methodologies that examine the associations between specific predictors and mental health outcomes in Bangladesh. Although informative, such approaches may overlook the heterogeneity in how mental health symptoms and risk factors cluster within individuals. For example, a recent study involving 1496 Bangladeshi adolescents reported that 15.6% had at least one current psychiatric disorder, including emotional disorders (9.1%), conduct problems (21.7%), hyperactivity (6.2%), and peer‐relationship difficulties (15.1%) (Al‐Mamun, Islam, et al. 2024). Among younger children aged 3–4 years, prevalence rates were lower, with 4.4% exhibiting emotional problems, 5.6% conduct disorders, and 1.9% hyperactivity symptoms (Mullick and Islam 2020).

Suicidal behavior represents another critical mental health concern. According to the 2014 Global School‐Based Student Health Survey, 11.7% of school‐going adolescents in Bangladesh reported experiencing suicidal behaviors in the past year, with 4.9% reporting suicidal ideation, 7.4% a suicide plan, and 6.7% a suicide attempt (Khan et al. 2020). A more recent study found that 6.8% of adolescents reported lifetime suicidal ideation, with 2.3% having formulated a plan and 0.8% reporting a suicide attempt. Corresponding past‐year rates were 3.2% for ideation, 1.5% for planning, and 0.6% for attempts (Al‐Mamun, Al Habib, Islam, et al. 2025). Additionally, a substantial proportion of adolescents face psychosocial stressors: A total of 10.9% reported loneliness in the past year, 24.5% experienced being bullied in the past month, and 30.9% engaged in truancy during the same period. Furthermore, 15.4% lacked peer support, 45.9% indicated that their parents rarely checked their homework, 52.4% reported poor understanding with their parents, and an alarming 91.5% reported having no close friends (Khan et al. 2020).

The present study is guided by a developmental psychopathology framework, which emphasizes how early life stress and relational adversities disrupt the development of emotion regulation and behavioral control systems, increasing the likelihood of mental health problems during adolescence (Cicchetti and Rogosch 2002). In line with Bronfenbrenner's ecological systems theory (1979), we also consider how individual vulnerabilities interact with microsystem‐level influences, such as parent–child communication, peer support, and household context, to shape adolescent psychological outcomes in resource‐constrained environments (Bronfenbrenner 1979).

Considering the complexity of the co‐occurring emotional and behavioral problems (EBPs) during the developmental period, latent class analysis (LCA) or latent profile analysis (LPA) have been increasingly used to explore heterogeneity among adolescents (Lanza and Cooper 2016). Findings from global studies using LCA or LPA identified patterns of psychosocial risk among adolescents both in high‐income (Bianchi et al. 2017; Eastman et al. 2018; Kretschmer et al. 2015; Luk et al. 2012) and LMIC settings (Fine et al. 2022; González‐Forteza et al. 2017; Ma et al. 2019; Zhao et al. 2019). For example, four latent classes were identified among adolescents (Bianchi et al. 2017; Kretschmer et al. 2015; Rong et al. 2025), whereas three classes were characterized in a sample from rural China (Zhao et al. 2019), and five classes were revealed by the Tibetan and Han adolescents (Ma et al. 2019). Despite this growing evidence base, few studies in Bangladesh have holistically examined the co‐occurrence of EBPs alongside psychosocial adversities such as insomnia, loneliness, school truancy, involvement in physical altercations, bullying, and lack of social connections. This gap highlights the need for person‐centered approaches, such as LCA, to better identify and characterize distinct mental health risk profiles among adolescents (Lanza and Cooper 2016), fostering early intervention efforts (Lanza and Rhoades 2013).

To address these gaps, the present study aimed to identify distinct mental health and behavioral risk profiles among Bangladeshi adolescents using a person‐centered analytic approach. LCA was employed to uncover subgroups based on co‐occurring EBP (e.g., emotional symptoms, conduct issues, hyperactivity, and peer problems), psychosocial stressors (e.g., insomnia, loneliness, truancy, bullying, and involvement in physical fights), and indicators of social isolation (e.g., lack of close friends). Building on this classification, a series of multinomial logistic regression models were estimated to examine how ACEs, family and peer environments, and sociodemographic characteristics predicted latent class membership. Particular attention was given to gender‐specific vulnerabilities, assessed through interaction terms between gender and ACEs. We hypothesized that adolescents exposed to greater childhood adversity, poorer family relationships, and limited peer support would be more likely to belong to higher risk psychosocial profiles. By integrating these analytic strategies, this study offers a nuanced understanding of the heterogeneity in adolescent mental health profiles and the complex interplay of psychosocial and contextual risk factors. The findings aim to inform the development of contextually tailored mental health screening tools, early intervention strategies, and prevention programs suited to adolescents in Bangladesh and comparable low‐resource settings.

## 2 Methods

### 2.1 Study Design, Settings, and Procedure

This cross‐sectional study was carried out between September and October 2024 among secondary school students in Phulbari, a sub‐district of Kurigram, Bangladesh. Phulbari was selected based on logistical feasibility and accessibility. A two‐stage stratified cluster sampling approach was utilized. In the first stage, schools were stratified by geographic location (urban vs. rural), and eight schools were randomly selected—three from urban and five from rural areas. In the second stage, students from Grades 7 through 10 in each selected school were targeted, with all students in these grades invited to participate.

Students were eligible to participate if they met the following criteria: (i) present during the day of the survey and (ii) enrolled in Grades 7–10. Exclusion criteria included (i) lack of informed consent, (ii) any disability that hindered participation, (iii) the absence on the day of data collection, and (iv) incomplete responses for the primary outcome variable.

Prior to initiating data collection, formal permission was obtained from school principals and classroom teachers. Written informed consent was required from both students and their parents or guardians. Upon receipt of signed consent forms, trained research staff administered the survey. During data collection, both teachers and members of the research team were available to assist students and clarify any questions regarding the survey instrument. A pilot test involving 30 students was conducted to assess the questionnaire's clarity and comprehensibility; however, data from the pilot were excluded from the final analysis. Finally, 1040 complete cases were included for the analyses.

### 2.2 Measures

#### 2.2.1 Sociodemographic Factors

The study collected data on key sociodemographic variables, including participants’ gender (male and female), age group (12–14 and 15–17 years), educational grade (7, 8, 9, and 10), family type (nuclear vs. joint), and geographical location (urban vs. rural). These variables were selected on the basis of their potential influence on psychosocial outcomes and were used as covariates in adjusted multinomial logistic regression models.

#### 2.2.2 Family and Peer Environment

Three binary items were used to evaluate recent familial and peer dynamics. Adolescents were asked whether their parents regularly checked their homework, whether they experienced poor understanding with their parents, and whether they lacked peer support. Each item referred to experiences within the past month and was coded as Yes (1) or No (0) (Khan et al. 2020).

#### 2.2.3 Adverse Childhood Experiences

ACEs were measured using a structured self‐report instrument encompassing multiple forms of childhood abuse and household dysfunction, as established in prior research (Felitti et al. 1998; Hughes et al. 2017). The abuse domain consisted of three subtypes: psychological, physical, and sexual abuse. Psychological abuse included verbal aggression or behaviors by a parent or household adult that instilled fear of physical harm. Physical abuse referred to incidents where the child was pushed, slapped, grabbed, or physically harmed in ways that could cause visible injury. Sexual abuse was assessed by asking about experiences of being inappropriately touched, fondled, or coerced into sexual acts. Household dysfunction was assessed through indicators, such as exposure to mental illness, domestic violence, and household criminality. Mental illness was defined as having a family member with a diagnosed psychiatric condition or a history of attempted suicide. Exposure to domestic violence was captured by reports of physical abuse directed at the respondent's mother, including acts like slapping, pushing, or threats involving weapons. Criminal behavior within the household was assessed by asking whether any family member had been incarcerated. A respondent was considered exposed to an ACE domain if they reported at least one affirmative response within that domain.

#### 2.2.4 Latent Class Indicators

To identify distinct subgroups of adolescents based on co‐occurring mental health symptoms and behavioral risks, 11 binary indicators were utilized in the LCA. Each variable was coded as 1 for symptom or behavior endorsement (presence) and 0 for non‐endorsement. These indicators were selected based on their theoretical and empirical relevance to adolescent psychological functioning and risk profiles.

Four indicators were derived from the Bengali version of the Strengths and Difficulties Questionnaire (SDQ), a widely used tool for assessing emotional and behavioral difficulties in youth. This version, available for noncommercial use from the official SDQ website [https://www.sdqinfo.org/py/sdqinfo/b3.py?language=Bengali], comprises five subscales: emotional symptoms (e.g., “often unhappy, downhearted”), conduct problems (e.g., “fights with other children”), hyperactivity/inattention (e.g., “constantly fidgeting or squirming”), peer relationship problems (e.g., “tends to play alone”), and prosocial behavior (e.g., “considerate of other people's feelings”). Each subscale contains five items rated on a 3‐point scale (0 = “not true,” 1 = “somewhat true,” 2 = “certainly true”). Following established guidelines, scores were dichotomized using clinical cutoffs: emotional symptoms ≥ 7, conduct problems ≥ 5, hyperactivity/inattention ≥ 7, and peer problems ≥ 6 (out of 10) (Goodman et al. 1998). Prosocial behavior was not included in the latent class indicators. The scale has been previously used in the Bangladeshi context (Al‐Mamun, Islam, et al. 2024; Mullick and Islam 2020).

Beyond SDQ‐based indicators, additional psychosocial and behavioral risk factors were included. Insomnia symptoms were assessed using a brief two‐item version of the insomnia severity index (ISI), covering satisfaction with current sleep patterns and the degree to which sleep difficulties interfered with daily functioning. Each item was rated on a 5‐point Likert scale (0 = “very satisfied” to 4 = “very dissatisfied”). A total score of ≥6 (range: 0–8) was used to identify clinically significant insomnia, based on established thresholds with a sensitivity of 84% and specificity of 76% (Kraepelien et al. 2021). The scale has been previously used in the Bangladeshi context (Al‐Mamun, Mamun, et al. 2024; Hasan et al. 2021).

Other mental health and behavioral risks included lifetime suicide attempts, past‐year loneliness, and school‐ or peer‐related behavioral problems (Al‐Mamun, Al Habib, Islam, et al. 2025; Khan et al. 2020). Suicidal behavior was assessed with a single item asking whether the respondent had ever attempted suicide (Al‐Mamun, Al Habib, Islam, et al. 2025). Loneliness was evaluated based on self‐reported experiences of feeling lonely during the past 12 months. Truancy was defined as skipping school without permission in the past month. Physical fighting was assessed by asking participants whether they had been involved in a fight over the past year. Experiences of bullying victimization in the past month were also recorded. Finally, participants were asked whether they had any close friends, with endorsement of “no close friends” coded as a psychosocial risk (Khan et al. 2020).

These 11 dichotomized indicators were entered into the LCA to empirically derive latent profiles representing varying levels of risk.

### 2.3 Ethical Consideration

All procedures followed the ethical principles outlined in the Declaration of Helsinki ‐ 1975, as revised in 2013. Ethical approval was granted by the CHINTA Research Bangladesh Ethics committee [Ref: chinta/2024/08(1)]. Institutional approvals were also obtained from school principals and teachers. Participation was voluntary, and written informed consent was obtained from both students and their parents or legal guardians. Students were assured of their right to withdraw at any point without any repercussions. No incentives or compensation were provided. These ethical safeguards ensured the study's adherence to standards of research integrity and participant protection.

### 2.4 Statistical Analysis

All analyses were conducted using R version 4.3.1. Descriptive statistics were computed to summarize the frequencies and percentages of sociodemographic characteristics, ACEs, and family environment factors. Missingness patterns were evaluated using *MissMech* package in R. Because the data violated multivariate normality assumptions, the nonparametric test of homoscedasticity was used to assess the missing completely at random (MCAR) assumption. The test indicated no significant evidence against MCAR (*p* = 0.658). Missingness across variables ranged from 0% to 5.35%. After listwise deletion, 1040 participants were retained in the final analytic sample. Given support for the MCAR assumption, complete‐case analysis using listwise deletion was considered appropriate.

#### 2.4.1 Latent Class Analysis

LCA was used to identify subgroups of adolescents with similar profiles of mental health symptoms and behavioral risks. Eleven binary indicators were selected, including four SDQ domains (emotional symptoms, conduct problems, hyperactivity/inattention, and peer problems), psychosocial stressors (e.g., insomnia, loneliness, truancy, bullying, and involvement in physical fights), and indicators of social isolation (e.g., lack of close friends). Each indicator was dichotomized to reflect presence (1) or absence (0) of the characteristic. Before running LCA, these variables were recoded to 1 = “No” and 2 = “Yes” as required by the poLCA package.

LCA models with 2–5 classes were estimated using maximum likelihood estimation with 5000 iterations. To reduce the likelihood of local maxima and improve solution stability, each latent model was estimated using 50 random starts. Model fit was compared using Bayesian information criterion (BIC), Akaike information criterion (AIC), adjusted BIC (aBIC), likelihood‐ratio chi‐square statistic (*G* ^2^), and relative entropy. Model selection was additionally guided by class size, interpretability, and convergence stability across random starts. Although the poLCA package does not provide Lo–Mendell–Rubin likelihood ratio testing or formal diagnostics for local independence, model selection incorporated complementary evaluation of model parsimony and stability. The optimal number of classes was determined primarily based on the lowest BIC, aBIC, and acceptable entropy. Class membership was assigned based on the highest posterior probability, and class labels (e.g., low risk, moderate risk, and high risk) were determined by the mean probability of symptom endorsement across indicators. Average posterior probabilities were additionally examined to assess classification quality.

#### 2.4.2 Bivariate Analysis

To examine differences in predictor variables across the identified latent classes, chi‐square tests of independence were performed using the rcompanion package. Effect sizes were reported using Cramér's *V*, with values interpreted as negligible (<0.10), small (0.10–0.29), medium (0.30–0.49), or large (≥0.50). Each categorical predictor (e.g., gender, family type, and ACEs) was cross‐tabulated with the latent class variable, and row‐wise percentages were calculated to facilitate interpretation.

#### 2.4.3 Multinomial Logistic Regression

A series of multinomial logistic regression models were conducted to identify factors associated with latent class membership among adolescents, with the “low‐risk” class serving as the reference category. These analyses were performed using the *nnet* package in R. A total of six hierarchical models were estimated sequentially to examine the incremental contribution of ACEs, sociodemographic characteristics, and family environment factors.

Model 1 included only ACE‐related predictors: psychological abuse, physical abuse, sexual abuse, household mental illness, mother was treated violently, and incarceration of a family member. In Model 2, demographic variables were added to the ACE predictors. These covariates included gender, age category, grade level, family type (nuclear vs. joint), and location (urban vs. rural), allowing assessment of whether demographic context accounted for variation in class membership.

Model 3 extended the previous model by incorporating family and peer environmental factors. These included parental monitoring of homework (past month), poor understanding with parents (past month), and lack of peer support (past month)—each of which may impact adolescent psychosocial adjustment and interact with earlier adversities.

To explore potential moderation by gender, Model 4 tested interaction effects between gender and each ACE variable, enabling assessment of whether the associations between specific childhood adversities and class membership varied by gender. Model 5 retained these interaction terms and adjusted for demographic covariates, whereas Model 6 added the family and peer environmental factors for full adjustment.

For all models, odds ratios (ORs), 95% confidence intervals (CIs), and *p* values were reported. Model fit was assessed using McFadden's pseudo *R* ^2^, as implemented in the *pscl* package. All statistical tests were two‐sided, with a significance level set at *p* < 0.05.

### 2.5 Artificial Intelligence Disclosure

Generative AI tools (ChatGPT and OpenAI) were used for language refinement and editing of the manuscript. The authors reviewed and approved all content and take full responsibility for the integrity and accuracy of the manuscript.

## 3 Results

### 3.1 Latent Class Model Selection and Fit Evaluation

Table 1 presents the fit statistics for latent class models with two to five class solutions based on mental health symptom indicators. Model selection was guided by a combination of statistical fit indices, parsimony, and substantive interpretability. Specifically, we considered the AIC, BIC, sample‐size aBIC, entropy, likelihood‐ratio goodness‐of‐fit statistic (*G* ^2^), and the proportion of individuals assigned to the smallest latent class.

**TABLE 1:** Fit indices for latent class models of mental health and behavioral risk profiles.

Classes	Log‐likelihood	Parameters	AIC	BIC	aBIC	*G* ^2^	Entropy	Smallest class (%)	Convergence stability
2	−4939.831	23	9925.663	10,039.440	9966.392	832.814	0.733	36.83	Stable across 50 starts
3	−4896.024	35	9862.047	10,035.190	9924.027	745.198	0.761	27.63	Stable across 50 starts
4	−4873.704	47	9841.409	10,073.920	9924.638	700.560	0.816	11.43	Multiple local maxima
5	−4858.240	59	9834.479	10,126.350	9938.959	669.630	0.851	1.12	Unstable

*Note: G* ^2^ = likelihood‐ratio goodness‐of‐fit statistic. Entropy values closer to 1 indicate better classification precision. Convergence stability was evaluated across 50 random starts.

Abbreviations: aBIC, sample‐size‐adjusted Bayesian information criterion; AIC, Akaike information criterion; BIC, Bayesian information criterion.

The five‐class model showed the lowest AIC (9834.47) and *G* ^2^ (669.63), indicating improved fit relative to the more parsimonious models. However, this model also produced a very small class (1.12% of the sample), raising concerns about overfitting and interpretability. Additionally, the five‐class model showed evidence of instability across random starts and multiple local maxima. The four‐class model similarly yielded a relatively low AIC (9841.41) but also included a small latent class (11.43%), which may limit its utility in applied contexts. This model likewise demonstrated reduced convergence stability relative to the more parsimonious solutions.

In contrast, the three‐class solution demonstrated a more optimal balance between statistical fit and conceptual clarity. This model yielded favorable aBIC (9924.03) and BIC (10,035.19) values compared to the two‐class model and avoided very small classes, with the smallest class comprising a substantial 27.63% of the sample. The model's entropy value (0.761) indicates moderate classification certainty, suggesting acceptable separation between classes. Importantly, the three‐class solution demonstrated stable convergence across all 50 random starts, supporting solution stability. Average posterior probabilities ranged from 0.750 to 0.804, indicating acceptable classification quality and moderate assignment precision across classes. Although the four‐ and five‐class models demonstrated slightly improved entropy values, these solutions showed evidence of instability across random starts and local maxima, reducing their interpretability and parsimony.

On the basis of these considerations, the three‐class model was retained as the most parsimonious and interpretable representation of latent heterogeneity in mental health symptom patterns. Class labels were assigned based on overall patterns of symptom and behavioral risk endorsement across indicators.

### 3.2 Conditional Response Probabilities and Latent Class Profiles

Table 2 displays the conditional probabilities of endorsing mental health symptoms and behavioral risk indicators across the three latent classes identified in the optimal model. These probabilities provide insight into the defining characteristics of each class and facilitate interpretation based on symptom severity and behavioral patterns.

**TABLE 2:** Conditional response probabilities across latent classes.

Variable	Low risk (382; 36.27%)	Moderate risk (381; 36.10%)	High risk (277; 27.63%)
Emotional problems	0.008	0.055	0.273
Conduct problems	0.067	0.101	0.584
Hyperactivity	0.011	0.016	0.206
Peer problems	0.036	0.141	0.301
Insomnia	0.028	0.044	0.098
Lifetime suicide attempt	0.016	0.010	0.087
Loneliness (past year)	0.108	0.046	0.262
Truancy (past month)	0.485	0.090	0.485
Physical fight involvement (past year)	0.503	0.040	0.623
Bullied (past month)	0.492	0.094	0.484
No close friends	0.653	0.546	0.521

*Note*: Values represent conditional probabilities of endorsing each indicator within each latent class. Higher values indicate greater likelihood of symptom or behavioral risk endorsement.

Class 1 (*n* = 382; 36.27%) showed low probabilities of SDQ‐based emotional and behavioral symptoms and, insomnia, with emotional problems (0.008), conduct problems (0.067), hyperactivity (0.011), peer problems (0.036), and insomnia (0.028) rarely reported. However, this class had relatively higher probabilities of some school‐ and peer‐related behavioral/social indicators, including physical fight involvement (0.503), bullying victimization (0.492), and truancy (0.485), and absence of close friends (0.653). Therefore, this class was interpreted as a comparatively lower clinical symptom group rather than uniformly “low‐risk” group across all psychological indicators.

Class 2 (*n* = 381; 36.10%) exhibited moderately elevated probabilities for internalizing and behavioral symptoms, including emotional problems (0.055), conduct problems (0.101), peer problems (0.141), and insomnia (0.044). Notably, this group showed the lowest levels of truancy (0.090) and physical fight involvement (0.040) among the classes, suggesting a distinct profile of mild emotional and interpersonal difficulties with relatively limited externalizing behaviors. This class was labeled the “moderate‐risk” group.

Class 3 (*n* = 277; 27.63%) demonstrated the highest probability of symptom endorsement across nearly all indicators. Emotional (0.273), conduct (0.584), hyperactivity (0.206), peer problems (0.301), insomnia (0.098), and loneliness (0.262) were substantially more prevalent in this group. Additionally, physical fighting (0.623) and bullying (0.484) were highly prevalent, alongside elevated emotional, conduct, and peer‐related difficulties, reflecting both internalizing and externalizing vulnerability. This class exhibited the greatest overall psychosocial vulnerability and behavioral risk burden and was therefore classified as the “high‐risk” group.

### 3.3 Descriptive Characteristics of the Study Sample

Table 3 presents the descriptive statistics of the study variables, highlighting key demographic characteristics, parenting context, and ACEs among Bangladeshi adolescents. The majority of participants were male (52.79%) and aged between 12 and 14 years (58.65%). Most students were enrolled in Grade 7 (32.40%) and resided in nuclear families (70.67%). Slightly more than half of the sample (51.92%) lived in urban areas. In terms of the family environment, a substantial proportion of adolescents reported poor understanding with their parents (72.88%) and that their parents rarely checked their homework (53.56%). Regarding peer relations, 37.31% of adolescents experienced a lack of peer support. Among the reported ACEs, psychological abuse was the most prevalent (19.13%), followed by household mental illness (18.08%) and having an imprisoned family member (8.37%).

**TABLE 3:** Descriptive characteristics of study variables.

Variable	Category	Frequency	Percentage
Gender	Male	549	52.79
	Female	491	47.21
Age category	12–14 years	610	58.65
	15–17 years	430	41.35
Grade	7	337	32.40
	8	316	30.38
	9	273	26.25
	10	114	10.96
Family type	Nuclear	735	70.67
	Joint	305	29.33
Location	Urban	540	51.92
	Rural	500	48.08
Parents rarely check homework (past month)	No	483	46.44
	Yes	557	53.56
Poor understanding with parents (past month)	No	282	27.12
	Yes	758	72.88
Lack of peer support (past month)	No	652	62.69
	Yes	388	37.31
Psychological abuse	No	841	80.87
	Yes	199	19.13
Physical abuse	No	959	92.21
	Yes	81	7.79
Sexual abuse	No	983	94.52
	Yes	57	5.48
Household mental illness	No	852	81.92
	Yes	188	18.08
Mother was treated violently	No	966	92.88
	Yes	74	7.12
Family member imprisoned	No	953	91.63
	Yes	87	8.37

### 3.4 Bivariate Associations Between Study Variables and Latent Class Membership

Table 4 displays the bivariate associations between individual‐level predictors and mental health risk classes derived from the LCA. Significant group differences in class membership were observed across several demographic, psychosocial, and adversity‐related variables.

**TABLE 4:** Bivariate associations between study variables and latent class membership.

Variable	Category	Low risk	Moderate risk	High risk	*χ* ^2^ (*p* value)	Cramér's *V*
Gender	Male	174; 31.7%	218; 39.7%	157; 28.6%	12.71 (0.0017)	0.111
	Female	208; 42.4%	163; 33.2%	120; 24.4%		
Age category	12–14	209; 34.3%	240; 39.3%	161; 26.4%	5.44 (0.0660)	0.072
	15–17	173; 40.2%	141; 32.8%	116; 27.0%		
Grade level	Grade 7	121; 35.9%	131; 38.9%	85; 25.2%	6.65 (0.3547)	0.057
	Grade 8	108; 34.2%	124; 39.2%	84; 26.6%		
	Grade 9	103; 37.7%	89; 32.6%	81; 29.7%		
	Grade 10	50; 43.9%	37; 32.5%	27; 23.7%		
Family type	Nuclear	273; 37.1%	262; 35.6%	200; 27.2%	1.10 (0.5779)	0.032
	Joint	109; 35.7%	119; 39.0%	77; 25.2%		
Location	Urban	173; 32.0%	204; 37.8%	163; 30.2%	12.45 (0.0020)	0.109
	Rural	209; 41.8%	177; 35.4%	114; 22.8%		
Parents rarely check HW	No	195; 40.4%	172; 35.6%	116; 24.0%	5.84 (0.0541)	0.075
	Yes	187; 33.6%	209; 37.5%	161; 28.9%		
Poor understanding with parents	No	115; 40.8%	80; 28.4%	87; 30.9%	11.53 (0.0031)	0.105
	Yes	267; 35.2%	301; 39.7%	190; 25.1%		
Lack of peer support	No	285; 43.7%	217; 33.3%	150; 23.0%	37.19 (<0.001)	0.189
	Yes	97; 25.0%	164; 42.3%	127; 32.7%		
Psychological abuse	No	337; 40.1%	301; 35.8%	203; 24.1%	24.49 (<0.001)	0.154
	Yes	45; 22.6%	80; 40.2%	74; 37.2%		
Physical abuse	No	370; 38.6%	350; 36.5%	239; 24.9%	25.11 (<0.001)	0.155
	Yes	12; 14.8%	31; 38.3%	38; 46.9%		
Sexual abuse	No	373; 37.9%	363; 36.9%	247; 25.1%	22.92 (<0.001)	0.148
	Yes	9; 15.8%	18; 31.6%	30; 52.6%		
Household mental illness	No	327; 38.4%	315; 37.0%	210; 24.6%	10.62 (0.0049)	0.101
	Yes	55; 29.3%	66; 35.1%	67; 35.6%		
Mother was treated violently	No	374; 38.7%	354; 36.6%	238; 24.6%	34.90 (<0.001)	0.183
	Yes	8; 10.8%	27; 36.5%	39; 52.7%		
Family member imprisoned	No	361; 37.9%	341; 35.8%	251; 26.3%	6.74 (0.0344)	0.080
	Yes	21; 24.1%	40; 46.0%	26; 29.9%		

*Note*: Values are presented as *n* (%); *χ* ^2^ = chi‐square statistic; *V* = Cramér's *V*.

Among sociodemographic variables, gender and location demonstrated significant associations with class membership. Males were more likely to belong to the moderate‐risk class (39.7%) compared to females (33.2%), whereas females showed a higher prevalence in the low‐risk group (42.4%) (*χ* ^2^ = 12.71, p = 0.001, Cramér's *V* = 0.111). Adolescents from rural areas were disproportionately represented in the low‐risk class (41.8%), whereas their urban counterparts had higher proportions in the high‐risk group (30.2%) (*χ* ^2^ = 12.45, p = 0.002, *V* = 0.109).

Indicators of family environment were also significantly related to mental health risk class. Adolescents who reported poor understanding with parents had higher representation in the moderate‐risk class (39.7%) compared to those with better parent–child understanding (28.4%) (*χ* ^2^ = 11.53, p = 0.003, *V* = 0.105). Lack of peer support was notably associated with higher risk; nearly one‐third (32.7%) of adolescents with poor peer support belonged to the high‐risk class compared to only 23.0% among those with sufficient peer support (*χ* ^2^ = 37.19, p < 0.001, *V* = 0.189).

Adolescents who experienced psychological, physical, or sexual abuse were more likely to belong to the high‐risk class. For instance, 52.6% of those who reported sexual abuse were classified as high risk compared to just 25.1% among those without such exposure (*χ* ^2^ = 22.92, p < 0.001, *V* = 0.148). Similar results were observed for psychological abuse (37.2% vs. 24.1%) and physical abuse (46.9% vs. 24.9%), all with statistically significant but small effect sizes. Household mental illness and exposure to maternal violence were also linked to increased high‐risk classification (35.6% and 52.7%, respectively), with the latter showing a small effect (*χ* ^2^ = 34.90, p < 0.001, *V* = 0.183). Having an incarcerated family member was also significantly associated with class membership, though the effect size was smaller (*χ* ^2^ = 6.74, p = 0.034, *V* = 0.080).

### 3.5 Childhood Adversity and Latent Class Membership

Multinomial logistic regression was conducted to examine the association between various forms of childhood adversity and adolescents’ likelihood of being classified into moderate‐ or high‐risk mental health profiles, with the low‐risk group serving as the reference category (Table 5).

**TABLE 5:** Multinomial logistic regression predicting latent class membership from childhood adversity.

Predictor	OR (moderate risk)	95% CI	*p* value	OR (high risk)	95% CI	*p* value
Intercept	0.81	[0.68, 0.96]	0.016	0.49	[0.40, 0.59]	<0.001
Psychological abuse (yes)	1.60	[1.05, 2.42]	0.027	1.76	[1.13, 2.74]	0.013
Physical abuse (yes)	1.78	[0.86, 3.67]	0.120	2.39	[1.15, 4.98]	0.020
Sexual abuse (yes)	1.41	[0.61, 3.27]	0.422	2.88	[1.29, 6.42]	0.010
Household mental illness (yes)	1.12	[0.75, 1.66]	0.589	1.56	[1.03, 2.37]	0.035
Mother was treated violently (yes)	2.23	[0.95, 5.25]	0.065	3.46	[1.49, 8.02]	0.004
Family member imprisoned (yes)	1.75	[1.00, 3.06]	0.050	1.27	[0.68, 2.39]	0.456

*Note*: Low‐risk class served as the reference category. Model fit indices: log‐likelihood (model): −1094.45; deviance (*G* ^2^): 74.40; McFadden's *R* ^2^: 0.033; McKelvey and Zavoina *R* ^2^: 0.069; Cox and Snell *R* ^2^: 0.078.

Abbreviations: CI, confidence interval; OR, odds ratio.

The results indicated that psychological abuse was significantly associated with increased odds of belonging to both the moderate‐risk (OR = 1.60, 95% CI [1.05, 2.42], p = 0.027) and high‐risk (OR = 1.76, 95% CI [1.13, 2.74], p = 0.013) classes, compared to the low‐risk group. Similarly, physical abuse was not significantly associated with moderate‐risk membership (p = 0.120), but it was a significant predictor of high‐risk status (OR = 2.39, 95% CI [1.15, 4.98], p = 0.020). Sexual abuse demonstrated a particularly strong association with high‐risk classification (OR = 2.88, 95% CI [1.29, 6.42], p = 0.010), though it was not a significant predictor of moderate risk (p = 0.422).

Among family dysfunction variables, exposure to household mental illness significantly increased the odds of high‐risk membership (OR = 1.56, 95% CI [1.03, 2.37], p = 0.035), but not moderate risk (p = 0.589). Adolescents who witnessed their mother being treated violently were over three times more likely to belong to the high‐risk group (OR = 3.46, 95% CI [1.49, 8.02], p = 0.004), with a marginal effect for the moderate‐risk class (p = 0.065). Additionally, having a family member imprisoned significantly predicted moderate‐risk class membership (OR = 1.75, 95% CI [1.00, 3.06], p = 0.050), though it was not associated with high risk (p = 0.456).

Model fit was acceptable, with a log‐likelihood of −1094.45 and a deviance statistic (*G* ^2^) of 74.40. Pseudo *R* ^2^ values indicated modest explanatory power (McFadden's *R* ^2^ = 0.033, Cox and Snell *R* ^2^ = 0.078, McKelvey and Zavoina *R* ^2^ = 0.069).

### 3.6 Childhood Adversity and Latent Class Membership Adjusted for Demographics

Multinomial logistic regression was conducted to evaluate whether childhood adversity predicts adolescents’ mental health profiles after adjusting for key demographic variables. The low‐risk class served as the reference category (Table 6).

**TABLE 6:** Multinomial logistic regression predicting latent class membership adjusted for demographic covariates.

Predictor	OR (moderate risk)	95% CI	*p* value	OR (high risk)	95% CI	*p* value
Intercept	1.24	[0.86, 1.78]	0.256	0.72	[0.47, 1.08]	0.110
Gender (female)	0.65	[0.49, 0.88]	0.006	0.71	[0.51, 0.99]	0.042
Age category (15–17 years)	0.69	[0.45, 1.06]	0.088	0.78	[0.49, 1.24]	0.294
Grade 8	1.14	[0.79, 1.66]	0.476	1.18	[0.77, 1.80]	0.445
Grade 9	1.02	[0.63, 1.67]	0.928	1.31	[0.77, 2.26]	0.322
Grade 10	0.95	[0.51, 1.79]	0.882	0.97	[0.48, 1.98]	0.942
Family type (joint)	1.11	[0.81, 1.53]	0.528	0.93	[0.65, 1.34]	0.696
Location (rural)	0.78	[0.59, 1.05]	0.104	0.67	[0.48, 0.93]	0.016
Psychological abuse (yes)	1.57	[1.03, 2.40]	0.038	1.63	[1.04, 2.56]	0.034
Physical abuse (yes)	1.59	[0.76, 3.30]	0.217	2.19	[1.04, 4.59]	0.038
Sexual abuse (yes)	1.36	[0.58, 3.17]	0.474	2.72	[1.22, 6.10]	0.015
Household mental illness (yes)	1.13	[0.75, 1.69]	0.553	1.63	[1.07, 2.49]	0.023
Mother was treated violently (yes)	2.17	[0.91, 5.14]	0.079	3.39	[1.45, 7.91]	0.005
Family member imprisoned (yes)	1.66	[0.94, 2.93]	0.079	1.29	[0.68, 2.44]	0.436

*Note*: Low‐risk class served as the reference category. Model fit: log‐likelihood = −1081.80; deviance (*G* ^2^) = 99.70; McFadden's *R* ^2^ = 0.044; McKelvey and Zavoina *R* ^2^ = 0.091; Cox and Snell *R* ^2^ = 0.103.

Abbreviations: CI, confidence interval; OR, odds ratio.

After controlling for gender, age, grade level, family type, and location, psychological abuse remained a significant predictor of both moderate‐risk (OR = 1.57, 95% CI [1.03, 2.40], p = 0.038) and high‐risk (OR = 1.63, 95% CI [1.04, 2.56], p = 0.034) membership. Similarly, physical abuse significantly predicted high‐risk class membership (OR = 2.19, 95% CI [1.04, 4.59], p = 0.038), though the association with moderate risk did not reach significance. Sexual abuse was again strongly associated with high‐risk classification (OR = 2.72, 95% CI [1.22, 6.10], p = 0.015), consistent with the unadjusted model.

Among the household adversity indicators, household mental illness was significantly associated with higher odds of high‐risk class membership (OR = 1.63, 95% CI [1.07, 2.49], p = 0.023). Exposure to maternal violence also significantly increased the likelihood of high‐risk classification (OR = 3.39, 95% CI [1.45, 7.91], p = 0.005), though its effect on moderate‐risk membership was not significant (p = 0.079).

Among demographic covariates, female gender was significantly associated with lower odds of belonging to both the moderate‐risk (OR = 0.65, 95% CI [0.49, 0.88], p = 0.006) and high‐risk (OR = 0.71, 95% CI [0.51, 0.99], p = 0.042) classes, suggesting that males were more likely to report elevated mental health symptoms. Adolescents residing in rural areas had significantly lower odds of high‐risk membership compared to their urban counterparts (OR = 0.67, 95% CI [0.48, 0.93], p = 0.016), indicating a possible urban vulnerability.

Fit statistics indicated improved model performance relative to the null model (*G* ^2^ = 99.70), with small‐to‐moderate effect sizes: McFadden's *R* ^2^ = 0.044, McKelvey and Zavoina *R* ^2^ = 0.091, and Cox and Snell *R* ^2^ = 0.103. These values suggest that childhood adversity, in conjunction with sociodemographic characteristics, contributes meaningfully to differentiating between adolescent mental health profiles.

### 3.7 Childhood Adversity and Latent Class Membership Adjusted for Demographics and Family Environment

Multinomial logistic regression was conducted to evaluate the association between childhood adversity and adolescents’ latent mental health risk profiles, adjusting for demographics and family environment factors. The low‐risk class served as the reference category (Table 7).

**TABLE 7:** Multinomial logistic regression predicting latent class membership adjusted for demographic and family environment variables.

Predictor	OR (moderate risk)	95% CI	*p* value	OR (high risk)	95% CI	*p* value
Intercept	0.64	[0.40, 1.03]	0.065	0.49	[0.29, 0.81]	0.006
Gender (female)	0.60	[0.45, 0.82]	0.001	0.67	[0.48, 0.94]	0.021
Age category (15–17 years)	0.66	[0.43, 1.02]	0.062	0.73	[0.46, 1.18]	0.205
Grade 8	1.13	[0.77, 1.65]	0.524	1.19	[0.78, 1.83]	0.418
Grade 9	1.10	[0.66, 1.81]	0.717	1.41	[0.81, 2.44]	0.224
Grade 10	0.95	[0.50, 1.81]	0.879	1.07	[0.52, 2.19]	0.863
Family type (joint)	1.15	[0.83, 1.59]	0.408	0.94	[0.65, 1.37]	0.763
Location (rural)	0.76	[0.56, 1.02]	0.066	0.64	[0.46, 0.90]	0.010
Parents rarely check homework (yes)	1.16	[0.86, 1.57]	0.321	1.33	[0.95, 1.85]	0.097
Poor understanding with parents (yes)	1.70	[1.20, 2.41]	0.003	1.04	[0.72, 1.49]	0.839
Lack of peer support (yes)	2.13	[1.55, 2.92]	<0.001	2.25	[1.59, 3.19]	<0.001
Psychological abuse (yes)	1.52	[0.98, 2.34]	0.059	1.51	[0.95, 2.39]	0.079
Physical abuse (yes)	1.49	[0.71, 3.16]	0.292	2.09	[0.99, 4.41]	0.054
Sexual abuse (yes)	1.43	[0.60, 3.37]	0.418	2.64	[1.16, 6.02]	0.021
Household mental illness (yes)	1.02	[0.68, 1.55]	0.911	1.47	[0.95, 2.25]	0.081
Mother was treated violently (yes)	2.29	[0.95, 5.50]	0.064	3.56	[1.51, 8.38]	0.004
Family member imprisoned (yes)	1.58	[0.89, 2.81]	0.120	1.23	[0.64, 2.35]	0.536

*Note*: Low‐risk class served as the reference category. Model fit: log‐likelihood = −1058.73; deviance (*G* ^2^) = 145.83; McFadden's *R* ^2^ = 0.064; McKelvey and Zavoina *R* ^2^ = 0.131; Cox and Snell *R* ^2^ = 0.148.

Abbreviations: CI, confidence interval; OR, odds ratio.

After full adjustment, lack of peer support remained a strong and consistent predictor of both moderate‐risk (OR = 2.13, 95% CI [1.55, 2.92], p < 0.001) and high‐risk (OR = 2.25, 95% CI [1.59, 3.19], p < 0.001) membership. Poor understanding with parents was significantly associated with moderate‐risk membership (OR = 1.70, 95% CI [1.20, 2.41], p = 0.003), though its effect on high‐risk membership was not significant.

Among childhood adversity indicators, sexual abuse remained a significant predictor of high‐risk membership (OR = 2.64, 95% CI [1.16, 6.02], p = 0.021), even after accounting for family environment and demographic covariates. The impact of mother being treated violently was also robust, showing a significant association with high‐risk class membership (OR = 3.56, 95% CI [1.51, 8.38], p = 0.004).

Regarding demographics, female adolescents were significantly less likely to be in either moderate‐risk (OR = 0.60, 95% CI [0.45, 0.82], p = 0.001) or high‐risk (OR = 0.67, 95% CI [0.48, 0.94], p = 0.021) groups compared to males, suggesting higher risk among boys. Similarly, adolescents from rural areas had significantly lower odds of being in the high‐risk class (OR = 0.64, 95% CI [0.46, 0.90], p = 0.010), consistent with findings in previous models.

Model diagnostics indicated further improvement in explanatory power compared to earlier models, with McFadden's *R* ^2^ = 0.064, McKelvey and Zavoina *R* ^2^ = 0.131, and Cox and Snell *R* ^2^ = 0.148. These values suggest that the combination of childhood adversity, demographic characteristics, and family environment explains a meaningful proportion of the variance in mental health risk classification among adolescents.

### 3.8 Gender Interactions Between Childhood Adversity and Latent Class Membership

To explore potential gender‐based differences in the impact of childhood adversity, a multinomial logistic regression model with interaction terms between gender and each adversity variable was estimated, using the low‐risk group as the reference category (Table 8).

**TABLE 8:** Multinomial logistic regression predicting latent class membership including gender × adversity interaction terms.

Predictor	OR (moderate risk)	95% CI	*p* value	OR (high risk)	95% CI	*p* value
Intercept	0.92	[0.72, 1.18]	0.511	0.62	[0.48, 0.82]	<0.001
Gender (female)	0.80	[0.57, 1.13]	0.201	0.60	[0.41, 0.90]	0.014
Psychological abuse (yes)	1.64	[0.96, 2.79]	0.070	1.47	[0.82, 2.63]	0.196
Physical abuse (yes)	1.73	[0.72, 4.14]	0.216	1.89	[0.76, 4.68]	0.168
Sexual abuse (yes)	2.26	[0.61, 8.39]	0.224	5.42	[1.52, 19.34]	0.009
Household mental illness (yes)	1.13	[0.66, 1.96]	0.652	1.01	[0.55, 1.84]	0.978
Mother treated violently (yes)	2.17	[0.67, 7.00]	0.194	4.29	[1.35, 13.61]	0.014
Family member imprisoned (yes)	2.44	[1.15, 5.19]	0.020	1.66	[0.71, 3.87]	0.238
Gender × psychological abuse	0.80	[0.33, 1.92]	0.615	1.44	[0.58, 3.58]	0.431
Gender × physical abuse	0.80	[0.16, 4.08]	0.790	1.87	[0.39, 9.05]	0.434
Gender × sexual abuse	0.42	[0.07, 2.49]	0.338	0.31	[0.05, 1.70]	0.176
Gender × household mental illness	0.86	[0.38, 1.95]	0.719	2.43	[1.05, 5.61]	0.037
Gender × mother was treated violently	1.31	[0.23, 7.52]	0.759	0.61	[0.11, 3.55]	0.586
Gender × family member imprisoned	0.37	[0.11, 1.23]	0.104	0.48	[0.13, 1.80]	0.274

*Note*: Low‐risk class served as the reference category. Model fit: log‐likelihood = −1082.42; deviance (*G* ^2^) = 98.45; McFadden's *R* ^2^ = 0.043; McKelvey and Zavoina *R* ^2^ = 0.090; Cox and Snell *R* ^2^ = 0.102.

Abbreviations: CI, confidence interval; OR, odds ratio.

Among the main effects, female gender was significantly associated with lower odds of belonging to the high‐risk class compared to males (OR = 0.60, 95% CI [0.41, 0.90], p = 0.014), whereas its association with moderate‐risk membership was not significant. Several adversity indicators retained significant main effects even after including interaction terms. Notably, sexual abuse was a robust predictor of high‐risk class membership (OR = 5.42, 95% CI [1.52, 19.34], p = 0.009), as was mother being treated violently (OR = 4.29, 95% CI [1.35, 13.61], p = 0.014). Additionally, having a family member imprisoned significantly increased the odds of being in the moderate‐risk class (OR = 2.44, 95% CI [1.15, 5.19], p = 0.020).

However, most interaction effects between gender and adversity variables were not statistically significant, indicating that the strength of association between childhood adversities and latent class membership did not differ substantially by gender. One exception was the interaction between gender and household mental illness, which showed a significant effect on high‐risk membership (OR = 2.43, 95% CI [1.05, 5.61], p = 0.037), suggesting that females exposed to household mental illness may face disproportionately higher psychological risk than their male counterparts.

Model diagnostics indicated modest improvement in model fit with the inclusion of interaction terms: McFadden's *R* ^2^ = 0.043, McKelvey and Zavoina *R* ^2^ = 0.090, and Cox and Snell *R* ^2^ = 0.102.

### 3.9 Gender Interactions Between Childhood Adversity and Latent Class Membership Adjustment for Demographics

To assess whether gender moderates the relationship between childhood adversity and risk group membership, a multinomial logistic regression model was estimated with interaction terms and demographic controls. The low‐risk class served as the reference category (Table 9).

**TABLE 9:** Multinomial logistic regression predicting latent class membership including gender × adversity interactions adjusted for demographics.

Predictor	OR (Mod. risk)	95% CI	*p* value	OR (high risk)	95% CI	*p* value
Intercept	1.15	[0.78, 1.68]	0.483	0.80	[0.50, 1.22]	0.300
Gender (female)	0.77	[0.54, 1.10]	0.148	0.60	[0.40, 0.90]	0.014
Psychological abuse (yes)	1.81	[1.05, 3.11]	0.032	1.52	[0.85, 2.74]	0.162
Physical abuse (yes)	1.60	[0.66, 3.84]	0.298	1.79	[0.72, 4.46]	0.212
Sexual abuse (yes)	2.21	[0.59, 8.26]	0.238	5.39	[1.51, 19.31]	0.010
Household mental illness (yes)	1.12	[0.64, 1.95]	0.687	1.02	[0.55, 1.88]	0.947
Mother treated violently (yes)	1.92	[0.59, 6.24]	0.280	3.96	[1.24, 12.67]	0.020
Family member imprisoned (yes)	2.53	[1.18, 5.42]	0.017	1.75	[0.75, 4.09]	0.195
Age category (15–17 years)	0.69	[0.45, 1.06]	0.091	0.79	[0.49, 1.27]	0.333
Grade 8	1.14	[0.79, 1.66]	0.477	1.14	[0.75, 1.74]	0.544
Grade 9	0.99	[0.61, 1.63]	0.984	1.22	[0.71, 2.11]	0.478
Grade 10	0.91	[0.48, 1.73]	0.783	0.95	[0.47, 1.93]	0.889
Family type (joint)	1.13	[0.82, 1.57]	0.443	0.91	[0.63, 1.31]	0.598
Location (rural)	0.78	[0.58, 1.05]	0.107	0.66	[0.47, 0.92]	0.014
Gender × psychological abuse	0.70	[0.29, 1.70]	0.426	1.24	[0.49, 3.11]	0.651
Gender × physical abuse	0.82	[0.16, 4.25]	0.817	1.94	[0.40, 9.48]	0.411
Gender × sexual abuse	0.42	[0.07, 2.54]	0.345	0.28	[0.05, 1.60]	0.152
Gender × household mental illness	0.90	[0.39, 2.04]	0.794	2.53	[1.09, 5.88]	0.032
Gender × mother was treated violently	1.62	[0.28, 9.41]	0.591	0.68	[0.12, 3.95]	0.667
Gender × family member imprisoned	0.33	[0.10, 1.13]	0.078	0.50	[0.13, 1.91]	0.308

*Note*: Low‐risk class served as the reference category. Model fit: log‐likelihood = −1073.01; deviance (*G* ^2^) = 117.27; McFadden's *R* ^2^ = 0.052; McKelvey and Zavoina *R* ^2^ = 0.107; Cox and Snell *R* ^2^ = 0.120.

Abbreviations: CI, confidence interval; OR, odds ratio.

After controlling for demographic covariates, several notable associations emerged. Female gender remained significantly associated with lower odds of high‐risk class membership (OR = 0.60, 95% CI [0.40, 0.90], p = 0.014), consistent with previous models, although the effect on moderate‐risk membership was not statistically significant.

Among adversity indicators, sexual abuse was a robust predictor of high‐risk membership (OR = 5.39, 95% CI [1.51, 19.31], p = 0.010), even after adjusting for demographic and gender interaction effects. Similarly, having a mother treated violently (OR = 3.96, 95% CI [1.24, 12.67], p = 0.020) and a family member imprisoned (OR = 2.53, 95% CI [1.18, 5.42], p = 0.017) were significantly associated with greater odds of moderate‐risk membership.

The interaction between gender and household mental illness was significant for the high‐risk class (OR = 2.53, 95% CI [1.09, 5.88], p = 0.032), suggesting that females exposed to mental illness in the household may experience heightened psychological vulnerability. No other interactions reached statistical significance.

Demographic covariates, such as rural residence, were again associated with lower odds of high‐risk membership (OR = 0.66, 95% CI [0.47, 0.92], p = 0.014), whereas other variables (age category, grade, and family type) did not significantly predict class membership.

Model fit indices indicated modest explanatory power, with McFadden's *R* ^2^ = 0.052, McKelvey and Zavoina *R* ^2^ = 0.107, and Cox and Snell *R* ^2^ = 0.120, slightly improved compared to prior models.

### 3.10 Gender Interactions Between Childhood Adversity and Latent Class Membership Adjustment for Demographics and Family Environment

In the fully adjusted model, several predictors retained significant associations with membership in the moderate‐ and high‐risk latent classes, compared to the low‐risk reference group (Table 10).

**TABLE 10:** Multinomial logistic regression predicting latent class membership including gender × adversity interaction terms adjusted for demographics and family environment.

Predictor	OR (Mod. risk)	95% CI	*p* value	OR (high risk)	95% CI	*p* value
Intercept	0.61	[0.37, 0.98]	0.041	0.54	[0.32, 0.91]	0.021
Gender (female)	0.72	[0.50, 1.03]	0.076	0.58	[0.39, 0.88]	0.011
Psychological abuse (yes)	1.83	[1.06, 3.17]	0.031	1.50	[0.83, 2.72]	0.181
Physical abuse (yes)	1.54	[0.63, 3.79]	0.345	1.76	[0.70, 4.45]	0.231
Sexual abuse (yes)	2.35	[0.61, 8.98]	0.212	5.22	[1.42, 19.10]	0.013
Household mental illness (yes)	1.03	[0.58, 1.81]	0.922	0.94	[0.50, 1.75]	0.841
Mother was treated violently (yes)	1.93	[0.59, 6.36]	0.280	3.98	[1.24, 12.83]	0.021
Family member imprisoned (yes)	2.30	[1.07, 4.95]	0.034	1.66	[0.75, 3.90]	0.249
Age category (15–17 years)	0.66	[0.42, 1.02]	0.060	0.74	[0.46, 1.20]	0.221
Grade 8	1.14	[0.78, 1.66]	0.508	1.16	[0.75, 1.78]	0.500
Grade 9	1.07	[0.65, 1.78]	0.784	1.32	[0.76, 2.31]	0.326
Grade 10	0.92	[0.48, 1.77]	0.812	1.04	[0.51, 2.15]	0.909
Family type (joint)	1.17	[0.84, 1.62]	0.352	0.92	[0.63, 1.33]	0.651
Location (rural)	0.75	[0.56, 1.02]	0.066	0.64	[0.45, 0.89]	0.008
Parents rarely check homework (yes)	1.15	[0.85, 1.55]	0.372	1.30	[0.93, 1.81]	0.129
Poor understanding with parents (yes)	1.65	[1.17, 2.34]	0.005	1.05	[0.73, 1.51]	0.803
Lack of peer support (yes)	2.18	[1.59, 3.01]	<0.001	2.23	[1.57, 3.17]	<0.001
Gender × psychological abuse	0.61	[0.24, 1.50]	0.280	1.05	[0.41, 2.67]	0.920
Gender × physical abuse	0.76	[0.14, 4.07]	0.750	1.78	[0.36, 8.82]	0.481
Gender × sexual abuse	0.39	[0.06, 2.44]	0.317	0.29	[0.05, 1.66]	0.163
Gender × household mental illness	0.87	[0.38, 2.02]	0.751	2.43	[1.03, 5.74]	0.042
Gender × mother was treated violently	1.78	[0.30, 10.53]	0.524	0.73	[0.12, 4.29]	0.727
Gender × family member imprisoned	0.38	[0.11, 1.32]	0.129	0.52	[0.13, 2.01]	0.341

*Note*: Low‐risk class served as the reference category. Model fit: log‐likelihood = −1050.72; deviance (*G* ^2^) = 161.85; McFadden's *R* ^2^ = 0.072; McKelvey and Zavoina *R* ^2^ = 0.144; Cox and Snell *R* ^2^ = 0.163.

Female gender was significantly associated with reduced odds of high‐risk class membership (OR = 0.58, 95% CI [0.39, 0.88], *p* = 0.011), whereas the association with the moderate‐risk class showed a nonsignificant trend (OR = 0.72, *p* = 0.076). This suggests female adolescents may be less likely to fall into the most severe symptom profile, even when adjusting for adversity and environment.

Among the childhood adversity variables, sexual abuse emerged as a strong and consistent predictor of high‐risk membership (OR = 5.22, 95% CI [1.42, 19.10], *p* = 0.013), even after adjusting for demographics, environment, and gender interactions. Mother being treated violently also significantly predicted high‐risk group membership (OR = 3.98, 95% CI [1.24, 12.83], *p* = 0.021), as did family member imprisonment for moderate‐risk class (OR = 2.30, 95% CI [1.07, 4.95], *p* = 0.034). Psychological abuse remained a significant predictor for the moderate‐risk class (OR = 1.83, *p* = 0.031), though its association with the high‐risk class was attenuated.

Poor understanding with parents (OR = 1.65, *p* = 0.005) was associated with moderate‐risk membership, whereas lack of peer support (ORs = 2.18 and 2.23 for moderate and high‐risk classes, respectively; both *p* < 0.001) was strongly associated with increased risk of both elevated symptom profiles. These findings emphasize the protective role of supportive family and peer relationships.

A significant interaction emerged for gender and household mental illness in predicting high‐risk membership (OR = 2.43, 95% CI [1.03, 5.74], *p* = 0.042). This indicates that the impact of household mental illness may be more pronounced among female adolescents in contributing to severe psychosocial risk. Other interaction terms were not statistically significant.

The overall model demonstrated improved explanatory power, with McFadden's *R* ^2^ = 0.072, McKelvey and Zavoina *R* ^2^ = 0.144, and Cox and Snell *R* ^2^ = 0.163, indicating that the inclusion of family environment and interaction terms enhanced model performance compared to previous models.

## 4 Discussion

This study identified three distinct mental health and behavioral risk profiles among Bangladeshi adolescents using LCA: low risk (36.3%), moderate risk (36.1%), and high risk (27.6%). The low‐risk class reported minimal SDQ‐based emotional or behavioral symptoms and insomnia, but still showed relatively high endorsement of some school‐ and peer‐related behavioral/social indicators; whereas the moderate‐risk class exhibited elevated internalizing symptoms (e.g., emotional‐ and peer‐related problems) but low levels of externalizing behaviors such as truancy or physical aggression. In contrast, the high‐risk class demonstrated co‐occurring internalizing and externalizing symptoms alongside higher rates of adversities, including loneliness, insomnia, bullying, and physical fights. Multinomial regression analyses consistently demonstrated that childhood adversities, particularly sexual abuse and exposure to maternal violence, were strong and significant predictors of membership in the high‐risk mental health profile. These associations remained robust even after adjusting for key demographic and family environment variables. In the fully adjusted model, lack of parental understanding and the absence of peer support were significant factors of moderate‐risk membership, whereas male gender, urban residence, sexual abuse, and maternal violence were associated with increased odds of being in the high‐risk group. These findings underscore the critical role of relational and contextual environments in shaping adolescent psychological vulnerability. Importantly, although female adolescents were generally less likely to be classified in the high‐risk group, a significant interaction between gender and household mental illness revealed that females exposed to mental illness within the household had disproportionately higher odds of severe psychological distress. This suggests a potential gender‐specific susceptibility to the psychological impacts of familial mental health conditions.

The identification of distinct latent classes reflecting co‐occurring emotional and behavioral symptoms aligns with prior person‐centered studies. Consistent with our findings, several studies have reported three‐class solutions comprising low‐risk, internalizing, and externalizing or comorbid symptom profiles. For instance, Zhao et al. (2019) identified three profiles among Chinese adolescents: an adequately adapted group, an internalizing problem group, and an externalizing problem group. Similarly, a multicountry analysis confirmed a comparable three‐class structure among Chinese adolescents, suggesting cross‐cultural consistency in latent mental health typologies during adolescence (Fine et al. 2022).

However, other studies have reported more differentiated class structures. A four‐class model was identified among Irish primary school children, including distinct profiles for emotional, behavioral, and combined emotional–behavioral difficulties, alongside a low‐risk group (Sharpe et al. 2023). Likewise, Rong et al. (2025) found four classes among adolescents: low‐symptom, emotional symptom, self‐harm, and high‐symptom, with the latter characterized by co‐occurring depression, anxiety, suicidal ideation, and non‐suicidal self‐injury. In a clinical sample of children with developmental coordination disorder, Pimenta et al. (2023) identified four classes: no mental health problems, hyperactivity, internalizing symptoms, and combined internalizing–externalizing problems. These differences in the number and nature of latent classes likely reflect variations in sample characteristics, age ranges, cultural context, and the breadth of mental health indicators included. The common emergence of a comorbid high‐risk group across studies reinforces the developmental psychopathology perspective that early life stress and relational adversity compromise emotion regulation and behavioral control systems, increasing the likelihood of co‐occurring mental health problems (Cicchetti and Toth 2008). These findings support the need for tailored intervention strategies that recognize both shared and context‐specific risk profiles among adolescents.

Moreover, the robust associations between childhood adversities, particularly sexual abuse and maternal exposure to violence, and elevated mental health risk reinforce the extensive literature documenting the long‐term impact of early interpersonal trauma on adolescent well‐being (Felitti et al. 1998; Hughes et al. 2017). In the present study, sexual abuse emerged as the strongest predictor of high‐risk class membership, consistent with prior research linking such experiences to a broad spectrum of mental health problems, including depression, anxiety, and behavioral dysregulation (Bentivegna and Patalay 2022; Khadr et al. 2018; Morais et al. 2018). Similarly, witnessing maternal violence was a significant predictor of severe psychosocial distress. This form of exposure is known to compromise adolescents’ emotional regulation and heighten vulnerability to both internalizing and externalizing psychopathologies (Airikka et al. 2023; Gourisankar et al. 2025; Peltonen et al. 2010; Silva et al. 2021). According to emotional security theory, chronic exposure to domestic violence fosters a perceived lack of safety and predictability in the home, which undermines emotional stability and increases the likelihood of maladaptive outcomes (Davies and Mark Cummings 1994). In collectivistic and family‐centered societies such as Bangladesh, where open expression of emotional distress may be culturally constrained, the psychological repercussions of domestic violence may be especially severe yet frequently overlooked. These findings emphasize the urgent need for trauma‐informed screening, prevention, and intervention strategies in youth mental health services. Early identification and support for adolescents exposed to family violence are essential to disrupting potential trajectories toward chronic mental health difficulties and social maladjustment.

In addition to childhood adversities, this study highlights the critical role of proximal relational environments, particularly family communication and peer support, in shaping adolescent mental health outcomes. Adolescents who reported poor parental understanding was associated with moderate‐risk membership, while lack of peer support was associated with both moderate‐ and high‐risk membership. These findings are consistent with earlier research identifying low family cohesion and weak peer connections as salient predictors of adolescent psychopathology (Butler et al. 2022; Nguyen et al. 2019; van Eickels et al. 2022). Supportive family interactions provide emotional scaffolding and protective buffering in the face of environmental stressors, whereas positive peer relationships serve as vital sources of identity development and belonging during adolescence (Butler et al. 2022; Demetriou 2025; Nguyen et al. 2019). The findings thus reaffirm that mental health interventions targeting adolescents must extend beyond individual symptomatology to consider broader interpersonal dynamics.

The gendered patterns observed in this study offer additional nuance to the understanding of adolescent mental health risk. Although females were generally less likely to belong to the high‐risk class, the significant interaction between gender and household mental illness revealed that girls exposed to mental illness in the home context may be disproportionately vulnerable to severe psychological distress. This finding aligns with prior evidence suggesting that female adolescents are more emotionally sensitive to family dysfunction and may internalize stressors to a greater extent than males (Al‐Mamun, Islam, et al. 2024; Davies and Lindsay 2004; Milas et al. 2025). Cultural expectations and gendered norms in South Asian contexts, including Bangladesh, may further compound this vulnerability by limiting girls’ access to emotional support, freedom of expression, or opportunities for help seeking. The broader implication is that although males may display higher rates of overt externalizing behaviors, leading to greater representation in higher risk classes, females exposed to familial stressors may suffer from more covert, yet equally impairing, forms of psychological distress that require tailored recognition and intervention.

## 5 Practical Implications

The findings of this study offer critical insights for adolescent mental health policy and practice in Bangladesh and similar low‐resource settings. The identification of a high‐risk group with co‐occurring internalizing and externalizing symptoms highlights the need for targeted screening tools that move beyond one‐size‐fits‐all models. Strong associations between childhood adversities (e.g., sexual abuse and maternal exposure to violence) and mental health risks underscore the urgency of trauma‐informed care, especially given cultural stigma around abuse and emotional distress. The importance of family communication and peer support suggests that interventions should incorporate parenting support and school‐based peer programs. Gender‐specific vulnerabilities, particularly among girls exposed to household mental illness, point to the need for gender‐responsive services, including safe spaces and mental health counseling. Integrating these strategies into national youth development plans and ensuring dedicated funding can promote adolescent well‐being and long‐term societal benefits.

## 6 Strength and Limitations

This study has several notable strengths. It is one of the first to use a person‐centered LCA approach to identify distinct mental health and behavioral risk profiles among adolescents in Bangladesh. By incorporating a broad range of emotional, behavioral, and psychosocial indicators along with family and contextual risk factors, the study provides a multidimensional view of adolescent psychological vulnerability. The inclusion of interaction terms in the multinomial regression models further allowed for the examination of gender‐specific effects, contributing to a more nuanced understanding of mental health disparities in low‐resource settings.

Despite these strengths, several limitations should be acknowledged. First, the cross‐sectional nature of the data limits causal inferences regarding the relationships between childhood adversities, family environment, and mental health outcomes. Second, the use of self‐reported measures may have introduced recall or social desirability biases, particularly in reporting sensitive issues, such as childhood abuse. Third, the sample consisted of in‐school adolescents only, potentially underrepresenting out‐of‐school youth who may face even greater mental health challenges. Additionally, the study was conducted in a single sub‐district of Bangladesh using a school‐based sample. Therefore, the findings may not be generalizable to all Bangladeshi adolescents, particularly those from other geographic, socioeconomic, or out‐of‐school populations. Regional differences in social, cultural, and educational contexts may influence adolescent mental health and behavioral risk patterns. Although missingness was consistent with an MCAR, the use of listwise deletion may have reduced statistical efficiency. Additionally, latent class models were estimated using the poLCA package in R, which does not provide certain advanced mixture‐model diagnostics available in software such as Mplus, including the Lo–Mendell–Rubin test and formal assessments of local independence. Finally, the entropy value of the selected latent class solution indicated moderate rather than excellent classification precision, suggesting that some degree of classification uncertainty may remain.

Future research should aim to replicate these findings using longitudinal designs to better understand the developmental pathways linking early adversity and relational environments with mental health outcomes. Including biological or clinical assessments (e.g., cortisol and diagnostic interviews) could strengthen the validity of mental health classification. Moreover, expanding the sample to include out‐of‐school or rural adolescents would improve generalizability. Finally, intervention research is needed to test the effectiveness of school‐based and family‐centered programs tailored to the specific needs of adolescents identified within high‐risk latent profiles.

## 7 Conclusion

This study provides novel insights into the heterogeneity of adolescent mental health in Bangladesh by identifying three distinct risk profiles through LCA. Results suggested the central role of childhood adversities, family communication, and peer support in shaping mental health outcomes. Particularly concerning is the elevated vulnerability among adolescents exposed to sexual abuse, maternal violence, and household mental illness, especially among females. These results call for the development of trauma‐informed, family‐ and peer‐based mental health interventions tailored to the needs of specific subgroups. Integrating these insights into national mental health strategies can help inform early identification, targeted prevention, and equitable service delivery for adolescents in Bangladesh and similar low‐resource settings.

## Author Contributions

**Firoj Al‐mamun**: conceptualization, methodology, validation, visualization, writing – original draft, writing – review and editing, formal analysis, data curation, project administration, investigation. **Moneerah Mohammad Almerab**: validation, visualization, supervision, writing – review and editing, methodology, resources, investigation. **Mohammed A. Mamun**: conceptualization, methodology, validation, visualization, writing – review and editing, data curation, project administration, investigation, supervision.

## Funding

Dr ALmerab is currently receiving funding support from the Princess Nourah Bint Abdulrahman University Researchers Supporting Project (Number PNURSP2026R563), Princess Nourah Bint Abdulrahman University, Riyadh, Saudi Arabia.

## Conflicts of Interest

The authors declare no conflicts of interest.

## Data Availability

The dataset associated with the manuscript is available from the corresponding author upon reasonable request.

## References

[brb371531-bib-0001] Airikka, A. , M. Lahti‐Pulkkinen , S. Tuovinen , et al. 2023. “Maternal Exposure to Childhood Maltreatment and Mental and Behavioral Disorders in Children.” European Child and Adolescent Psychiatry 32, no. 12: 2463–2475. 10.1007/S00787-022-02090-8. 36181574 PMC10682113

[brb371531-bib-0002] Al‐Mamun, F. , A. Al Habib , M. J. Abedin , M. M. Almerab , P. A. Atroszko , and M. A. Mamun . 2025. “Associations of adverse Childhood Experiences With Mental Health and Behaviors Among Adolescents.” BMC Public Health 25, no. 1: 4066. 10.1186/S12889-025-25246-Y. 41267029 PMC12632140

[brb371531-bib-0003] Al‐Mamun, F. , A. Al Habib , J. Islam , M. M. ALmerab , M. A. Mamun , and M. Muhit . 2025. “Lifetime and Past‐Year Suicidal Behaviors Among Adolescents in Bangladesh: A Two‐Stage Stratified Cluster Sampling Study.” Cambridge Prisms: Global Mental Health 12: e24. 10.1017/gmh.2025.10. 40028391 PMC11867828

[brb371531-bib-0004] Al‐Mamun, F. , J. Islam , M. Muhit , and M. A. Mamun . 2024. “Prevalence of Emotional and Behavioral Problems Among Adolescents in Bangladesh.” Social Psychiatry and Psychiatric Epidemiology 59, no. 12: 2215–2225. 10.1007/s00127-024-02673-7. 38684517

[brb371531-bib-0005] Al‐Mamun, F. , M. A. Mamun , M. E. Hasan , M. M. Almerab , and D. Gozal . 2024. “Exploring Sleep Duration and Insomnia Among Prospective University Students: A Study With Geographical Data and Machine Learning Techniques.” Nature and Science of Sleep 16: 1235–1251. 10.2147/NSS.S481786. PMC11344553 39184950

[brb371531-bib-0006] Bentivegna, F. , and P. Patalay . 2022. “The Impact of Sexual Violence in Mid‐Adolescence on Mental Health: A UK Population‐Based Longitudinal Study.” *The* Lancet Psychiatry 9, no. 11: 874–883. 10.1016/S2215-0366(22)00271-1. 36206779 PMC9630148

[brb371531-bib-0007] Bianchi, V. , P. Brambilla , M. Garzitto , et al. 2017. “Latent Classes of Emotional and Behavioural Problems in Epidemiological and Referred Samples and Their Relations to DSM‐IV Diagnoses.” European Child & Adolescent Psychiatry 26, no. 5: 549–557. 10.1007/S00787-016-0918-2. 27844161 PMC5394137

[brb371531-bib-0008] Bronfenbrenner, U. 1979. The Ecology of Human Development. Harvard University Press. https://www.hup.harvard.edu/books/9780674224575.

[brb371531-bib-0009] Butler, N. , Z. Quigg , R. Bates , et al. 2022. “The Contributing Role of Family, School, and Peer Supportive Relationships in Protecting the Mental Wellbeing of Children and Adolescents.” School Mental Health 14, no. 3: 776–788. 10.1007/S12310-022-09502-9. 35154501 PMC8818094

[brb371531-bib-0010] Cicchetti, D. , and F. A. Rogosch . 2002. “A Developmental Psychopathology Perspective on Adolescence.” Journal of Consulting and Clinical Psychology 70, no. 1: 6–20. 10.1037//0022-006X.70.1.6. 11860057

[brb371531-bib-0011] Cicchetti, D. , and S. L. Toth . 2008. “A Developmental Psychopathology Perspective on Adolescent Depression.” In Handbook of Depression in Adolescents, edited by S. Nolen‐Hoeksema and L. Hilt , 3–31. Taylor and Francis. 10.4324/9780203809518-8.

[brb371531-bib-0012] Davies, P. T. , and E. M. Cummings . 1994. “Marital Conflict and Child Adjustment: An Emotional Security Hypothesis.” Psychological Bulletin 116, no. 3: 387–411. 10.1037/0033-2909.116.3.387. 7809306

[brb371531-bib-0013] Davies, P. T. , and L. L. Lindsay . 2004. “Interparental Conflict and Adolescent Adjustment: Why Does Gender Moderate Early Adolescent Vulnerability?” Journal of Family Psychology 18, no. 1: 160–170. 10.1037/0893-3200.18.1.160. 14992618

[brb371531-bib-0014] Demetriou, C. 2025. “Family Functioning and Adolescents' Mental Health Problems: A Mixed‐Methods Analysis of Community and Clinical Samples.” International Journal of Developmental Science 19, no. 1–2: 5–15. 10.1177/2192001X251326198.

[brb371531-bib-0015] Eastman, M. , V. Foshee , S. Ennett , et al. 2018. “Profiles of Internalizing and Externalizing Symptoms Associated With Bullying Victimization.” Journal of Adolescence 65, no. 1: 101–110. 10.1016/J.ADOLESCENCE.2018.03.007. 29573643 PMC5932115

[brb371531-bib-0016] Felitti, V. J. , R. F. Anda , D. Nordenberg , et al. 1998. “Relationship of Childhood Abuse and Household Dysfunction to Many of the Leading Causes of Death in Adults: The Adverse Childhood Experiences (ACE) Study.” American Journal of Preventive Medicine 14, no. 4: 245–258. 10.1016/S0749-3797(98)00017-8. 9635069

[brb371531-bib-0017] Fine, S. L. , R. W. Blum , J. K. Bass , et al. 2022. “A Latent Class Approach to Understanding Patterns of Emotional and Behavioral Problems Among Early Adolescents Across Four Low‐ and Middle‐Income Countries.” Development and Psychopathology 35, no. 4: 1684–1700. 10.1017/S0954579422000384. 35635213 PMC9708939

[brb371531-bib-0018] González‐Forteza, C. , C. E. Juárez‐López , A. Jiménez , L. Montejo‐León , U. R. Rodríguez‐Santisbón , and F. A. Wagner . 2017. “Suicide Behavior and Associated Psychosocial Factors Among Adolescents in Campeche, Mexico.” Preventive Medicine 105: 206–211. 10.1016/J.YPMED.2017.09.011. 28917950

[brb371531-bib-0019] Goodman, R. , H. Meltzer , and V. Bailey . 1998. “The Strengths and Difficulties Questionnaire: A Pilot Study on the Validity of the Self‐Report Version.” European Child & Adolescent Psychiatry 7, no. 3: 125–130. 10.1007/s007870050057. 9826298

[brb371531-bib-0020] Gourisankar, A. , P. Ravi , A. Kalokhe , et al. 2025. “Examining the Impact of Maternal Experiences of Domestic Violence on the Mental Health of Their Adolescent Children in India.” PLoS ONE 20, no. 5: e0304936. 10.1371/JOURNAL.PONE.0304936. 40434972 PMC12118828

[brb371531-bib-0021] Hasan, M. , Z. Maliha , A. Rahman , and M. A. Mamun . 2021. “Insomnia in Bangladeshi Young Adults During the COVID‐19 Pandemic: The Role of Behavioral Factors, COVID‐19 Risk and Fear, and Mental Health Issues.” Sleep and Vigilance 5, no. 2: 315–322. 10.1007/S41782-021-00161-5. 34423233 PMC8366484

[brb371531-bib-0022] Hughes, K. , M. A. Bellis , K. A. Hardcastle , et al. 2017. “The Effect of Multiple Adverse Childhood Experiences on Health: A Systematic Review and Meta‐Analysis.” Lancet Public Health 2, no. 8: e356–e366. 10.1016/S2468-2667(17)30118-4. 29253477

[brb371531-bib-0023] Khadr, S. , V. Clarke , K. Wellings , et al. 2018. “Mental and Sexual Health Outcomes Following Sexual Assault in Adolescents: A Prospective Cohort Study.” *The* Lancet Child & Adolescent Health 2, no. 9: 654–665. 10.1016/S2352-4642(18)30202-5. 30119759

[brb371531-bib-0024] Khan, M. M. A. , M. M. Rahman , M. R. Islam , M. Karim , M. Hasan , and S. S. Jesmin . 2020. “Suicidal Behavior Among School‐Going Adolescents in Bangladesh: Findings of the Global School‐Based Student Health Survey.” Social Psychiatry and Psychiatric Epidemiology 55, no. 11: 1491–1502. 10.1007/s00127-020-01867-z. 32239265

[brb371531-bib-0025] Kieling, C. , C. Buchweitz , A. Caye , et al. 2024. “Worldwide Prevalence and Disability From Mental Disorders Across Childhood and Adolescence: Evidence From the Global Burden of Disease Study.” JAMA Psychiatry 81, no. 4: 347–356. 10.1001/jamapsychiatry.2023.5051. 38294785 PMC10831630

[brb371531-bib-0026] Kraepelien, M. , K. Blom , E. Forsell , et al. 2021. “A Very Brief Self‐Report Scale for Measuring Insomnia Severity Using Two Items From the Insomnia Severity Index—Development and Validation in a Clinical Population.” Sleep Medicine 81: 365–374. 10.1016/J.SLEEP.2021.03.003. 33813233

[brb371531-bib-0027] Kretschmer, T. , E. D. Barker , J. K. Dijkstra , A. J. Oldehinkel , and R. Veenstra . 2015. “Multifinality of Peer Victimization: Maladjustment Patterns and Transitions From Early to Mid‐Adolescence.” European Child & Adolescent Psychiatry 24, no. 10: 1169–1179. 10.1007/S00787-014-0667-Z/METRICS. 25540123

[brb371531-bib-0028] Lanza, S. T. , and B. R. Cooper . 2016. “Latent Class Analysis for Developmental Research.” Child Development Perspectives 10, no. 1: 59–64. 10.1111/CDEP.12163. 31844424 PMC6914261

[brb371531-bib-0029] Lanza, S. T. , and B. L. Rhoades . 2013. “Latent Class Analysis: An Alternative Perspective on Subgroup Analysis in Prevention and Treatment.” Prevention Science 14, no. 2: 157–168. 10.1007/S11121-011-0201-1. 21318625 PMC3173585

[brb371531-bib-0030] Luk, J. W. , J. Wang , and B. G. Simons‐Morton . 2012. “The Co‐Occurrence of Substance Use and Bullying Behaviors Among U.S. Adolescents: Understanding Demographic Characteristics and Social Influences.” Journal of Adolescence 35, no. 5: 1351–1360. 10.1016/J.ADOLESCENCE.2012.05.003. 22698675 PMC3432689

[brb371531-bib-0031] Ma, C. , Y. Ma , Y. Wang , and X. Lan . 2019. “Empathy and Psychosocial Adjustment in Tibetan and Han Adolescents: A Person‐Centered Approach.” Frontiers in Psychology 10: 470965. 10.3389/FPSYG.2019.01896. PMC6700380 31456727

[brb371531-bib-0032] Milas, G. , M. Ribar , and F. Ćavar . 2025. “Why Are Adolescent Girls More Prone to Stress‐Induced Depression? Testing Moderation, Mediation, and Reciprocal Causality in a Three‐Wave Longitudinal Study.” Journal of Research on Adolescence 35, no. 1: e70015. 10.1111/JORA.70015. 40028811 PMC11874174

[brb371531-bib-0033] Morais, H. B. , A. A. Alexander , R. L. Fix , and B. R. Burkhart . 2018. “Childhood Sexual Abuse in Adolescents Adjudicated for Sexual Offenses: Mental Health Consequences and Sexual Offending Behaviors.” Sexual Abuse 30, no. 1: 23–42. 10.1177/1079063215625224. 26792116

[brb371531-bib-0034] Mullick, M. S. I. , and M. Islam . 2020. “The Prevalence of Psychiatric Disorders Among 3–4 Year Olds in an Urban Sample in Bangladesh.” Asian Journal of Psychiatry 54: 102368. 10.1016/j.ajp.2020.102368. 33271689

[brb371531-bib-0035] Nguyen, H. T. L. , K. Nakamura , K. Seino , and S. Al‐Sobaihi . 2019. “Impact of Parent‐Adolescent Bonding on School Bullying and Mental Health in Vietnamese Cultural Setting: Evidence From the Global School‐Based Health Survey.” BMC Psychology 7, no. 1: 1–10. 10.1186/S40359-019-0294-Z. 30885261 PMC6421663

[brb371531-bib-0036] Patton, G. C. , S. M. Sawyer , J. S. Santelli , et al. 2016. “Our Future: A Lancet Commission on Adolescent Health and Wellbeing.” *The* Lancet 387, no. 10036: 2423–2478. 10.1016/S0140-6736(16)00579-1. 27174304 PMC5832967

[brb371531-bib-0037] Peltonen, K. , N. Ellonen , H. B. Larsen , and K. Helweg‐Larsen . 2010. “Parental Violence and Adolescent Mental Health.” European Child & Adolescent Psychiatry 19, no. 11: 813–822. 10.1007/S00787-010-0130-8. 20821263

[brb371531-bib-0038] Pimenta, R. A. , C. Fuchs , N. E. Fears , M. Mariano , and P. Tamplain . 2023. “Distinct Mental Health Profiles in Children With Developmental Coordination Disorder: A Latent Class Analysis and Associations.” Research in Developmental Disabilities 132: 104377. 10.1016/J.RIDD.2022.104377. 36423431

[brb371531-bib-0039] Rathod, S. , N. Pinninti , M. Irfan , et al. 2017. “Mental Health Service Provision in Low‐ and Middle‐Income Countries.” Health Services Insights 10: 1178632917694350. 10.1177/1178632917694350. 28469456 PMC5398308

[brb371531-bib-0040] Rong, F. , M. Wang , C. Peng , et al. 2025. “Aggression and Patterns of Co‐Occurrence Mental Health Problems in Chinese Adolescents: A Latent Class Analysis.” BMC Public Health 25, no. 1: 1–12. 10.1186/S12889-024-21136-X. 39754091 PMC11697733

[brb371531-bib-0041] Sharpe, J. , B. Bunting , and C. Heary . 2023. “A Latent Class Analysis of Mental Health Symptoms in Primary School Children: Exploring Associations With School Attendance Problems.” School Mental Health 15, no. 4: 1128–1144. 10.1007/S12310-023-09610-0.

[brb371531-bib-0042] Sheikh, M. A. , B. Abelsen , and J. A. Olsen . 2016. “Clarifying Associations Between Childhood Adversity, Social Support, Behavioral Factors, and Mental Health, Health, and Well‐Being in Adulthood: A Population‐Based Study.” Frontiers in Psychology 7: 727. 10.3389/fpsyg.2016.00727. 27252668 PMC4879780

[brb371531-bib-0043] Silva, E. P. , A. Emond , and A. B. Ludermir . 2021. “Depression in Childhood: The Role of Children's Exposure to Intimate Partner Violence and Maternal Mental Disorders.” Child Abuse & Neglect 122: 105305. 10.1016/J.CHIABU.2021.105305. 34517271

[brb371531-bib-0044] van Eickels, R. L. , A. Tsarpalis‐Fragkoulidis , and M. Zemp . 2022. “Family Cohesion, Shame‐Proneness, Expressive Suppression, and Adolescent Mental Health—A Path Model Approach.” Frontiers in Psychology 13: 921250. 10.3389/FPSYG.2022.921250. 35992453 PMC9382198

[brb371531-bib-0045] Zhao, J. , Q. Li , L. Wang , L. Lin , and W. Zhang . 2019. “Latent Profile Analysis of Left‐Behind Adolescents' Psychosocial Adaptation in Rural China.” Journal of Youth and Adolescence 48, no. 6: 1146–1160. 10.1007/S10964-019-00989-1. 30835034

